# Intestinal *PTGS2* mRNA Levels, *PTGS2* Gene Polymorphisms, and Colorectal Carcinogenesis

**DOI:** 10.1371/journal.pone.0105254

**Published:** 2014-08-28

**Authors:** Lotte K. Vogel, Mona Sæbø, Helle Høyer, Tine Iskov Kopp, Ulla Vogel, Sine Godiksen, Franz B. Frenzel, Julian Hamfjord, Inger Marie Bowitz-Lothe, Egil Johnson, Elin H. Kure, Vibeke Andersen

**Affiliations:** 1 Department of Cellular and Molecular Medicine, University of Copenhagen, Copenhagen, Denmark; 2 Department of Environmental and Health Studies, Telemark University College, Telemark, Norway; 3 Department of Laboratory Medicine, Telemark Hospital, Skien, Norway; 4 National Food Institute, Technical University of Denmark, Søborg, Denmark; 5 National Research Centre for the Working Environment, Copenhagen, Denmark; 6 Department of Genetics, Oslo University Hospital, Oslo, Norway; 7 Department of Pathology, Oslo University Hospital, Oslo, Norway; 8 Department of Gastrointestinal and Pediatric Surgery, Oslo University Hospital, Oslo, Norway; 9 Institute of Clinical Medicine, University of Oslo, Oslo, Norway; 10 Organ Center, Hospital of Southern Jutland, Aabenraa, Denmark; 11 Institute of Regional Health Research, University of Southern Denmark, Odense, Denmark; 12 Medical Department, Regional Hospital Viborg, Viborg, Denmark; Baylor College of Medicine, United States of America

## Abstract

**Background & Aims:**

Inflammation is a major risk factor for development of colorectal cancer (CRC). Prostaglandin synthase cyclooxygenase-2 (COX-2) encoded by the *PTGS2* gene is the rate limiting enzyme in prostaglandin synthesis and therefore plays a distinct role as regulator of inflammation.

**Methods:**

*PTGS2* mRNA levels were determined in intestinal tissues from 85 intestinal adenoma cases, 115 CRC cases, and 17 healthy controls. The functional *PTGS2* polymorphisms A-1195G (rs689466), G-765C (rs20417), T8473C (rs5275) were assessed in 200 CRC cases, 991 adenoma cases and 399 controls from the Norwegian KAM cohort.

**Results:**

*PTGS2* mRNA levels were higher in mild/moderate adenoma tissue compared to morphologically normal tissue from the same individual (P<0.0001) and (P<0.035) and compared to mucosa from healthy individuals (P<0.0039) and (P<0.0027), respectively. In CRC patients, *PTGS2* mRNA levels were 8–9 times higher both in morphologically normal tissue and in cancer tissue, compared to healthy individuals (P<0.0001). *PTGS2* A-1195G variant allele carriers were at reduced risk of CRC (odds ratio (OR) = 0.52, 95% confidence interval (95% CI): 0.28–0.99, P = 0.047). Homozygous carriers of the haplotype encompassing the A-1195G and G-765C wild type alleles and the T8473C variant allele *(PTGS2* AGC) were at increased risk of CRC as compared to homozygous carriers of the *PTGS2* AGT (A-1195G, G-765C, T8473C) haplotype (OR = 5.37, 95% CI: 1.40–20.5, P = 0.014). No association between the investigated polymorphisms and *PTGS2* mRNA levels could be detected.

**Conclusion:**

High intestinal *PTGS2* mRNA level is an early event in colorectal cancer development as it occurs already in mild/moderate dysplasia. *PTGS2* polymorphisms that have been associated with altered *PTGS2* mRNA levels/COX-2 activity in some studies, although not the present study, were associated with colorectal cancer risk. Thus, both *PTGS2* polymorphisms and *PTGS2* mRNA levels may provide information regarding CRC risk.

## Introduction

Colorectal cancer (CRC) constitutes the second most common cancer and the second most common cause of cancer-related deaths [1]. In the Western World, one in 20 will develop CRC before the age of 75 years [2]. Furthermore, the prevalence is increasing worldwide due to demographic trends and adaption to westernized lifestyle in developing countries [1]. Thus, identification of underlying biological mechanisms is of high importance in order to develop new preventive and treatment strategies.

Multiple environmental and genetic factors are involved in CRC [2], and intestinal inflammation has been found to be a major risk factor [3]–[6]. Cycloxygenase enzymes COX-1 and COX-2 encoded by the *PTGS1* and *PTGS2* genes catalyse the rate limiting step in the prostaglandin synthesis, which is a key regulator of inflammation. The *PTGS1* gene is constitutively expressed in many cell types, whereas the expression of the *PTGS2* gene is controlled by pro-inflammatory and mitogenic stimuli. Several polymorphisms in *PTGS2* that influence COX-2 enzyme levels have been described. The variant G-allele of *PTGS2* A-1195G destroys a c-Myb binding site in the promoter region resulting in lowered *PTGS2* mRNA levels in oesophageal tissue [7]. The *PTGS2* G-765 C polymorphism is located in a Stimulatory protein 1 (SP1) binding site. The G-765C C-allele had significantly 30% lower promoter activity compared with the G-allele in lung tissue [8]. In accordance with this higher COX-2 levels were found in the normal duodenal mucosa of patients with familial adenomatous polyposis who were homozygous carriers of the G-allele, compared to carriers of the C-allele [9]. On the other hand, no statistically significant differences between -765C and -765G were observed in transient reporter gene transfection studies in HeLa cells and no statistically significant difference was found in oesophageal tissue [7]. The *PTGS2* T8473C SNP is located in the 3′untranslated region of the *PTGS2* mRNA. Through binding at the 8473 3′UTR site, Mir-542-3p targets *PTGS2* mRNA for decay. The variant C-allele at 8473 disrupts miRNA-mRNA interaction leading to increased half-life of the *PTGS2* mRNA [10]. Carriers of the variant alleles of these SNPs thus have a genetically determined altered level of *PTGS2 mRNA* in tissues where the transcription factor or miRNA in question are expressed and functional.

High COX-2 levels have been found in a large proportion of adenoma and carcinoma tissues by immunohistochemistry [11], [12]. It is, however, not clear whether increased COX-2 expression occurs early in the adenoma-carcinoma sequence, thereby promoting carcinogenesis or whether it occurs late, possibly as a consequence of the carcinogenesis. In this study we assessed the levels of *PTGS2* mRNA in intestinal tissue from healthy subjects and adenoma and carcinoma cases using the Norwegian KAM cohort [13]–[24]. Tissue from the cases ranged from morphologically normal intestinal tissue to adenomas and carcinomas. We also assessed the potential risk of CRC associated with functional *PTGS2* gene polymorphisms. Furthermore, we assessed the association between the genetic variants in *PTGS2* and *PTGS2* mRNA levels.

## Materials and Methods

### Study cohort

The KAM (Kolorektal cancer, Arv og Miljø) cohort is based on the screening group of the Norwegian Colorectal Cancer Prevention study (the NORCCAP study) in the county of Telemark and a series of clinical CRC cases operated at Telemark Hospital (Skien) and Ullevaal University Hospital (Oslo) [15], [25]. In short, 20,780 healthy men and women, age 50–64 years of age, drawn at random from the population registry in Oslo (urban) and the county of Telemark (mixed urban and rural) were invited to have a flexible sigmoidoscopy screening examination.

The KAM biobank consists of samples from individuals with adenomas in the large intestine (991 adenomas and 53 hyperplastic polyps), 234 cases with adenocarcinomas and 400 controls, defined as individuals with normal findings at flexible sigmoidoscopy screening. The study was performed in accordance with the Helsinki Declaration. The Regional Ethics Committee and the Data Inspectorate approved the KAM study (S-98190, 2009/2021). The ID number for the study is NCT00119912 at ClinicalTrials.gov [26]. Written and verbal consent was obtained from all participants.

### Biological Material

Blood samples were available from 200 cases with CRC, 991 cases with adenomas and 399 controls and intestinal tissue was available from 115 cases with adenocarcinoma, 85 cases with adenomas and 17 healthy individuals [14]–[24]. From individuals with adenomas, control tissue was sampled 30 cm above the anus. From patients with carcinomas, two control samples were taken from the surgically specimen. One sample was taken adjacent to the cancer (normal adjacent) and the other sample was taken as distant from the cancer as possible (normal distant). Matching samples were available from 74 cases with mild-moderate dysplasia, 9 cases with severe dysplasia, and 93 (distant normal tissue samples) and 103 (adjacent tissue samples) CRC cases, respectively. The histology of the adenomas was examined independently by two pathologists, who categorised the degree of dysplasia as either mild/moderate (n = 76) or severe (n = 9). Consensus was reached in all cases. In a few cases biopsies of material suspected to be dysplastic were after examination of the histology categorized as normal and were classified as biopsies from healthy individuals (n = 17). Carcinomas were classified according to Dukes staging. Characteristics of the study population are shown in Table 1.

**Table 1:** Study participant description.

Genotyping study				
	Controls	Adenomas		Carcinomas
No. of subjects (N)	399	991		200
Male (N (%))	157 (39.3)	607 (61.2)		110 (55.0)
Female (N (%))	242 (60.7)	384 (38.8)		90 (45.0)
Age Mean (SD)	54.2 (3.3)	57.2 (3.7)		67.4 (11.2)
**mRNA study**				
	**Controls**	**Adenomas**		**Carcinomas**
		**Mild/moderate**	**Severe**	
No. of subjects (N)	17	76	9	115
Male (N (%))	5 (31)	52 (68)	4 (44)	64 (56)
Female (N (%))	11 (69)	24 (32)	5 (56)	51 (44)
Age Mean (SD)	57.2 (4.7)	56.8 (3.8)	55.4 (2.9)	69.8 (11.4)

### Real-time reverse transcriptase polymerase chain reaction

The tissue samples were frozen as soon as possible after surgery and stored in liquid nitrogen until RNA purification. Total RNA and cDNA synthesis was purified from tissue as described [19]. Quantitative real time RT-PCR of *PTGS2* was performed on the ABI7300 sequence detection system (Applied Biosystems) in Universal PCR Master Mix (part.no 4326614, Applied Biosystems) using 125 nM probe and 600 nM primers. Primers and probe were: *COX-2* forward 5′-ATT GTA CCC GGA CAG GAT TCT ATG -3′; *COX-2* reverse 5′-TTT GGA GTG GGT TTC AGA AAT AAT T-3′; *COX-2* probe 5′- FAM-CTG CTC AAC ACC GGA ATT TTT GAC AAG AAT-BHQ-3′.

Primers were designed within different exons and with the probe covering an exon-exon border to prevent amplification of genomic DNA. Primers and probes were obtained from TAG Copenhagen (Denmark). The endogenous *β-actin* control was obtained pre-developed (part.no.4310881E) from Applied Biosystems. In a validation experiment using a control sample, a dilution series was assayed by the comparative C_t_ method [27]. The assays were quantitative over a range of 128-fold dilution. Samples were quantified in triplicates. The CV of triplicates was 0.06 or less. The CV of repeated measurements of the same sample (the control) in separate experiments was 0.25, indicating the day-to-day variation of the assay. Negative controls (where the RNA was not converted into cDNA) and positive controls were included in all runs. Samples for which either the *β-actin* or *PTGS2* values fell outside the upper or lower limits of the standard curve were excluded from the study.

### Genotyping

Genomic DNA was isolated from blood samples according to standard procedures with minor modifications as described previously [28]. All analyses were run blinded to the case-control status. *PTGS2 T8473C* (rs5275) was genotyped using the following primers *PTGS2* FP 5′-GCA TCT TCC ATG ATG CAT TAG AAG TAA C-3′; *PTGS2* RP 5′-GGT AAT GTC TAA TTT AAA TAT TCA TTT AAT AAT GCA CTG ATA CC-3′; Probe for T allele 5′-FAM-ACT TTT GGT **T**AT TTT TC-MGB-3′; Probe for C allele 5′-VIC-CTT TTG GT**C** ATT TTT C-MGB3’. Controls were included in each run and repeated genotyping of a random 10% subset yielded 100% identical genotypes. *PTGS2* G-765C (rs20417) and *PTGS2* A-1195G (rs689466) were genotyped by KBioscience (kbioscience.co.uk). Genotype distributions of the polymorphisms among the controls did not deviate from Hardy-Weinberg equilibrium. Haplotypes were inferred manually.

### Statistics

Minitab 16 was used for the statistical analysis of the association between genotypes and risk of adenomas and risk of colorectal cancer adjusted for age and gender. All statistical analyses of mRNA levels were performed using SAS (release 9.3, SAS Institute, Cary, NC). Linear regression (PROC GLM) was used to compare mRNA levels in tissue from healthy participants versus tissues from affected participants with adjustment for age and gender. A paired t-test (PROC TTEST) was used to compare mRNA levels from control tissue and affected tissue from the same individual. All values of mRNA expression levels were log-transformed to correct for left-skewed distribution.

## Results

### *PTGS2* mRNA levels in intestinal tissue

**Figure 1:**
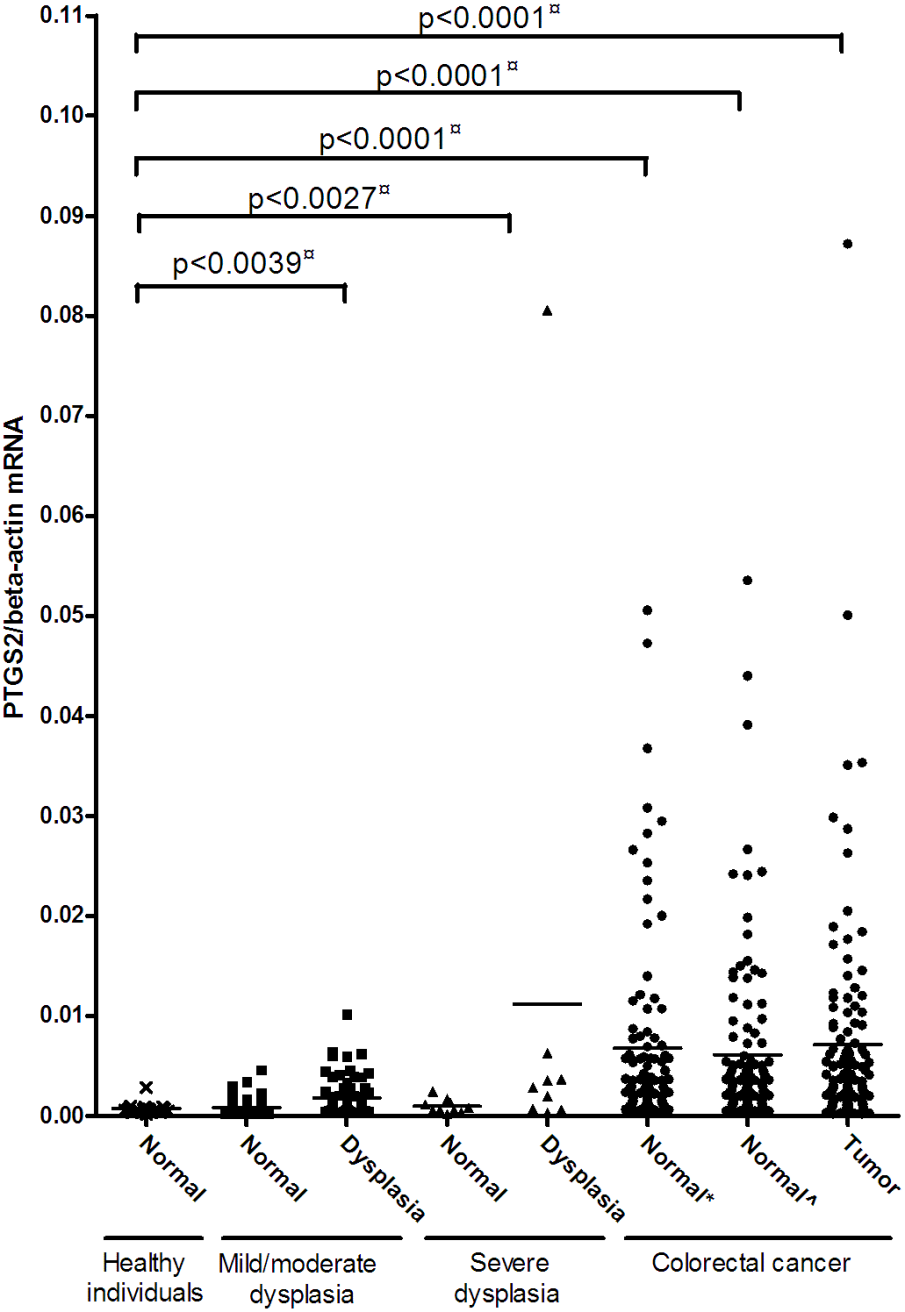
*PTGS2* mRNA expression during colorectal carcinogenesis. Normalised *PTGS2* mRNA levels in healthy individuals, individuals with mild/moderate dysplasia, severe dysplasia, and individuals with carcinomas as determined by real-time RT-PCR. Samples from healthy individuals (cross), normal and affected tissue from individuals with mild/moderate dysplasia (square), normal and affected tissue from individuals with severe dysplasia (triangle), and normal adjacent, normal distant and cancerous tissue from colorectal cancer patients (circle) were analyzed for *PTGS2* mRNA levels relative to the β-actin mRNA levels. The horizontal line represents the mean values. The p-value indicated with a ¤ was calculated using linear regression to compare mRNA levels in tissue from healthy participants versus tissues from affected participants with adjustment for age and gender.

*PTGS2* mRNA levels were increased in both mild/moderate and severe dysplastic tissue and in morphologically normal and affected tissue from cancer patients. *PTGS2* mRNA levels were significantly higher in mild/moderate dysplasia (P<0.0039), in severe dysplasia (P<0.0027), in morphologically normal mucosa from cancer patient, in both distant and adjacent to the tumour (P<0.0001 for both) and in tumour tissues (P<0.0001) as compared to the mucosa from healthy individuals (Figure 1 and Table 2). *PTGS2* mRNA levels were on an average almost 9 fold higher in cancerous tissue compared to tissue from healthy individuals. When comparing normal and affected tissue from the same individual, a statistically significant difference was seen in individuals with mild/moderate dysplasia (P<0.0001) and severe dysplasia (P<0.035) (Table 2). There was an inverse correlation between age and *PTGS2* mRNA levels (Figure S1). No correlation between the *PTGS2* mRNA levels and gender or Duke’s stage of the carcinoma (Dukes stage A (n = 19), stage B (n = 47), and stage C (n = 29)) was found (data not shown).

**Table 2:** *PTGS2* mRNA levels in morphologically normal and affected tissues normalised to the *β-actin* mRNA level.

	Normal Tissue		Adenoma/Carcinoma Tissue		
	Mean ± S.D.	P^a^	Mean ± S.D.	P^a^	P^b^
Healthy individuals	0.00072±0.00061				
Individuals with mild/moderate dysplasia	0.00083±0.00074	0.17	0.0018±0.0018	0.0039	<0.0001
Individuals with severe dysplasia	0.00096±0.00070	0.32	0.011±0.026	0.0027	0.035
Cancer patients	0.0068±0.0097 (distant)	<0.0001			0.98
			0.0071±0.011	<0.0001	
	0.0061±0.0088 (adjacent)	<0.0001			0.64

a P values for comparison to healthy individuals adjusted for age and gender.

b P value for comparison to morphologically normal tissue from the same individual using Paired Student’s T-test.

### Associations between *PTGS2* polymorphisms and CRC

Carriers of the *PTGS2* A-1195G variant G-allele were at lower risk of CRC (OR = 0.52, 95% CI: 0.28–0.99, P = 0.047) (Table 3). Furthermore, in a haplotype analysis, homozygous carriers of the haplotype encompassing the *PTGS2* T8473C variant allele (AGC) were at increased risk of CRC (OR = 5.37, 95% CI: 1.40–20.5, P = 0.014) compared to homozygous carriers of the reference *PTGS2* AGT (A-1195G, G-765C, T8473C) haplotype (Table 4).

**Table 3:** Risk estimates for the studied *PTGS2* polymorphisms in relation to risk of colorectal adenomas and carcinomas.

Genotypes		Controls	Cases Adenomas			Cases Carcinomas		
		N	N	OR (95% CI)^1^	P-value	N	OR (95% CI)^1^	P-value
A-1195G (rs689466)	AA	209	626	1		110	1	
	AG	114	284	0.89 (0.66–1.19)	0.428	24	0.55 (0.28–1.06)	0.072
	GG	11	23	0.50 (0.22–1.14)	0.097	2	0.34 (0.05–2.38)	0.277
	AG+GG	125	307	0.85 (0.63–1.13)	0.262	26	0.52 (0.28–0.99)	0.047
*G-765C* (rs20417)	GG	279	689	1		103	1	
	GC	84	207	1.05 (0.76–1.45)	0.750	30	0.86 (0.42–1.73)	0.669
	CC^2^	1	11	-	-	3	-	-
	GC+CC	85	218	1.11 (0.81–1.52)	0.532	33	0.96 (0.48–1.89)	0.897
*T8473C* (rs5275)	TT	169	413	1		69	1	
	TC	191	455	1.00 (0.76–1.31)	0.975	87	0.93 (0.55–1.57)	0.782
	CC	39	115	1.29 (0.83–2.00)	0.262	33	1.76 (0.81–3.82)	0.150
	TC+CC	230	570	1.05 (0.81–1.35)	0.74	120	1.06 (0.64–1.75)	0.820

1 Odds ratio (95% confidence intervals). All risk estimates are adjusted for age and gender.

2 Too few for calculation.

**Table 4:** Risk estimates for *PTGS2* haplotypes in relation to risk of colorectal adenomas and carcinomas.

	Controls	Cases Adenomas				Cases Carcinomas			
	N	N	OR^1^	(95% CI)	P-value^2^	N	OR	(95% CI)	P-value
AGT/AGT	70	200	REF		1	23	1		1
AGT/GGT	56	154	0.94	(0.60–1.48)	0.786	15	1.17	(0.41–3.34)	0.769
AGT/AGC	65	186	0.96	(0.62–1.48)	0.845	25	1.08	(0.39–2.96)	0.888
AGT/ACC	32	106	1.23	(0.72–2.09)	0.448	13	2.03	(0.64–6.43)	0.230
GGT/GGT	11	22	0.58	(0.24–1.41)	0.227	2	-	-	-
GGT/AGC	33	68	0.90	(0.52–1.56)	0.709	6	-	-	-
GGT/ACC	19	44	0.91	(0.47–1.76)	0.773	3	-	-	-
AGC/AGC	10	36	1.09	(0.48–2.48)	0.840	12	5.37	(1.40–20.5)	0.014
AGC/ACC	18	45	0.90	(0.46–1.79)	0.771	11	1.32	(0.35–4.91)	0.683
ACC/ACC	1	11	-	-	-	3	-	-	-

Haplotype sequence: *PTGS2* A-1195G (rs689466), G-765C (rs20417), T8473C (rs5275).

1 Odds ratio, 95% confidence interval.

2 Adjusted for age and gender.

### *PTGS2* gene polymorphisms and *PTGS2* mRNA levels

**Figure 2:**
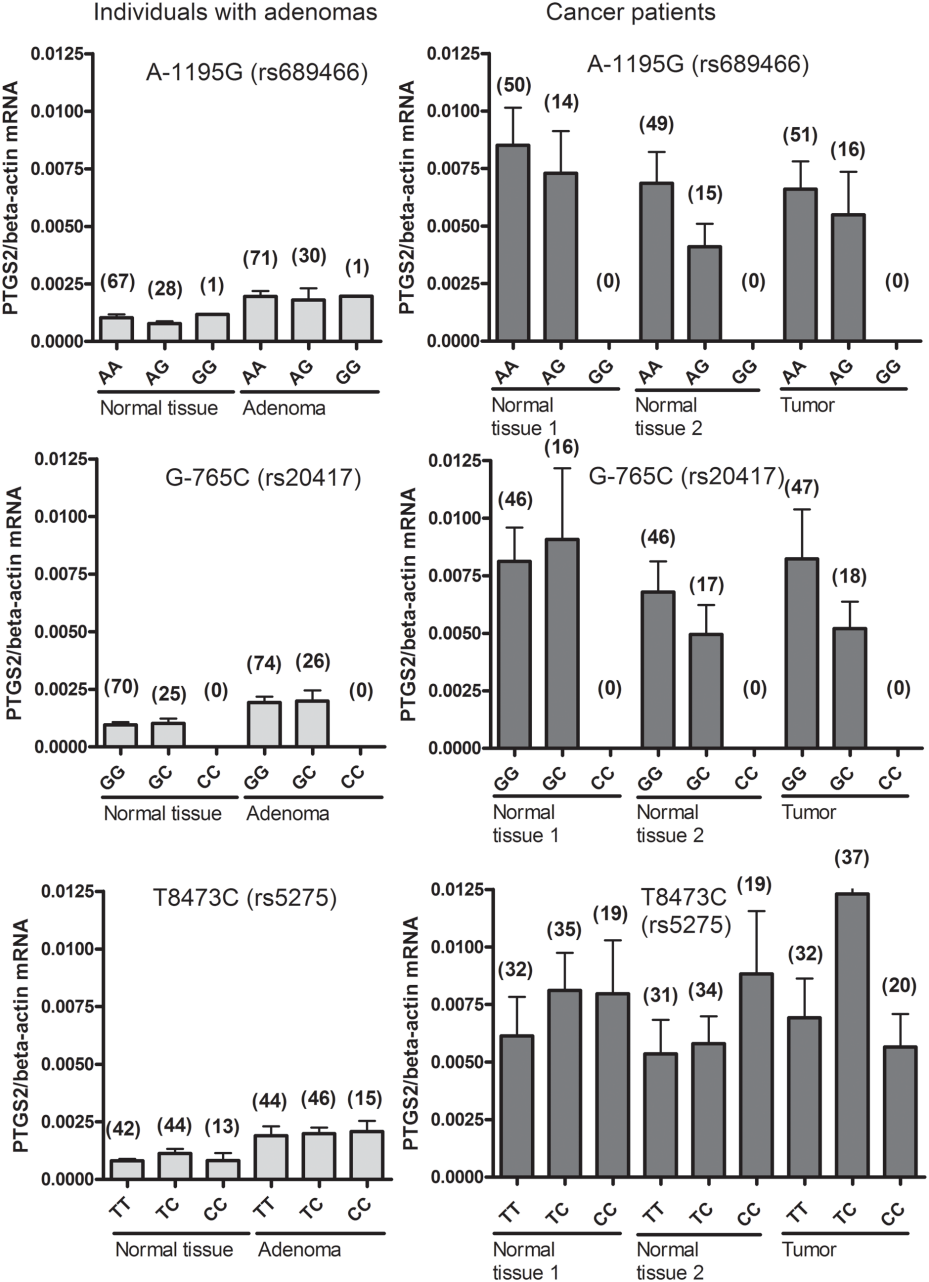
*PTGS2* gene polymorphisms and *PTGS2* mRNA level. Normalised *PTGS2* mRNA levels in morphologically normal and affected intestinal tissue from individuals with adenomas (left panel) and CRC (right panel) subdivided by *PTGS2* A-1195G (rs689466), G-765C (rs20417), T8473C (rs5275) genotypes. The number of individuals with each genotype is indicated in brackets above the column.

*PTGS2* mRNA levels in intestinal adenoma cases (left panel) and carcinoma cases (right panel) were subdivided by *PTGS2* A-1195G (rs689466), *PTGS2* G-765C (rs20417), and *PTGS2* T8473C (rs5275) genotypes, respectively (Figure 2). No statistically significant associations between genotypes and *PTGS2* mRNA levels were found (Figure 2).

## Discussion

The role of COX-2 in colorectal cancer carcinogenesis is not clear. We have therefore determined the *PTGS2* mRNA levels during colorectal cancer carcinogenesis and found that the mRNA level of *PTGS2* is increased already in mild/moderate dysplasia and in severe dysplasia as compared to morphologically normal tissue from the same individual and as compared to normal mucosa from healthy individuals. Furthermore, the *PTGS2* mRNA levels were increased both in normal and affected tissue from colorectal cancer patients compared to tissue from healthy control individuals. This is in accordance with previous investigations where COX-2 enzyme was detected in colorectal adenoma and carcinoma tissue by immunohistochemistry but was absent from normal tissue from healthy individuals and normal tissue from individuals with dysplasia [11], [12]. This indicates that high COX-2 expression occurs as an early event in colorectal cancer carcinogenesis.

The role of *PTGS2* in colorectal carcinogenesis was also studied by assessing the association of three functional polymorphisms in *PTGS2* with risk of development of adenoma and CRC. The *PTGS2* A-1195G variant G-allele and the *PTGS2* G-765C variant C-allele lead to lower transcription of the *PTSG2* gene. Conversely, the *PTGS2* T8473C variant C-allele leads to higher mRNA levels due to impaired mRNA degradation. The *PTGS*2 A-1195G variant G-allele co-segregates with the wild-type alleles of the two other polymorphisms, whereas the *PTGS2* G-765C variant C- allele and T8473C variant C-allele are in tight linkage [29]. The allele frequency of the T8473C variant C- allele is much higher than the allele frequency of the G-765C variant C-allele [30], [31].

We found that *PTGS2* A-1195G G- variant allele carriers were at reduced risk of CRC. We were unable to find any association between *PTGS2* A-1195G genotype and *PTGS2* mRNA levels, although analysis of the normal and affected tissue from cancer patients showed a tendency towards *PTGS2* A-1195G variant G-allele carriers having a lower mean level of *PTGS2* mRNA compared to individuals homozygous for the wild-type allele.

Due to the linkage of the SNPs most individuals carry a mixture of SNPs predisposing for low *PTGS2* mRNA level and SNPs predisposing for high *PTGS2* mRNA level. However, individuals homozygous for the haplotype encompassing *PTGS2* T8473C variant allele (A-1195G, G-765C, T8473C) (AGC) only carry alleles predisposing for high *PTGS2* mRNA levels. Homozygous carriers of the haplotype encompassing *PTGS2* T8473C variant allele (A-1195G, G-765C, T8473C) (AGC) were at 5-fold increased risk of CRC (P = 0.014) compared to homozygous carriers of the reference *PTGS2* AGT haplotype. In the present study we were unable to detect an altered risk of cancer for carriers of the G-765C variant C-allele, probably due to the strong linkage to T8473C variant C-allele, an allele with the opposite effects on the *PTGS2* mRNA level. Since the variant alleles of A-1195G and T8473C are present on different haplotypes, the two results point to the same conclusion, namely that high *PTGS2* mRNA level is associated with increased risk of CRC. Thus, results obtained in the present study suggest that the genotypes at *PTSG2* A-1195G and T8473C affect the risk of cancer. However, we were unable to demonstrate an altered *PTGS2* mRNA level depending on the genotype at position -1195 and 8473. Our finding correlate well with a meta-analysis finding that homozygosity for the *PTGS2-*1195 G-allele is associated with reduced risk of digestive system cancers [32]. However, the validity of this meta-analysis is unclear as we noted that for several of the papers analysed, the allele frequencies of G- and A-alleles of *PTGS2* A-1195G do not match the allele frequencies in the original papers [33], [34]. Our results are also in accordance with another meta-analysis showing that there is no evidence that the genotype at *PTGS2-*765 influences risk of colorectal cancer except in populations of Asian descent [35]. However, the results obtained in the present study are in contrast with results from the Danish prospective Diet, Cancer and Health cohort, where we found that high *PTGS2* mRNA level-associated *PTGS2* variant alleles were associated with lower risk of CRC [36]. This may be explained by effect modification of associations between the *PTGS2* SNP and colorectal cancer risk by dietary factors that may differ between Danish and Norwegian populations as for example fish and fruit and vegetables [36].

Our results suggest that high *PTGS2* expression is an early event in CRC carcinogenesis. Long term use of aspirin and other NSAIDs including selective COX-2 inhibitors have been associated with lowered risk of CRC [37], [38]. Also the present study and previous meta-analyses suggest that the genotypes at *PTGS2* A-1195G and T8473C but not G-765C are associated with altered risk of colorectal cancer, although we were unable to demonstrate association to altered levels of *PTGS2* mRNA levels for any of the three SNPs. This may suggest that the polymorphisms influence risk in other ways than by affecting *PTGS2* transcription levels.

## Conclusion

In conclusion, this study suggests that increased *PTGS2* mRNA level is an early event in colorectal cancer carcinogenesis. *PTGS2* SNPs that have been associated with altered *PTGS2* mRNA levels/COX-2 activity in some studies, although not the present study, were associated with colorectal cancer risk. Thus, both *PTGS2* polymorphisms and *PTGS2* mRNA levels may provide information regarding CRC risk.

## Supporting Information

Figure S1 **Normalised** ***PTGS2*** **mRNA levels decreased with age in normal tissue from individuals with dysplasia.**
(TIF)
