# Supplementary figures and images for: Intestinal PTGS2 mRNA Levels, PTGS2 Gene Polymorphisms, and Colorectal Carcinogenesis
Source: PLoS One. 2014 Aug 28;9(8):e105254. doi: 10.1371/journal.pone.0105254 (PMC4148233; doi:10.1371/journal.pone.0105254)

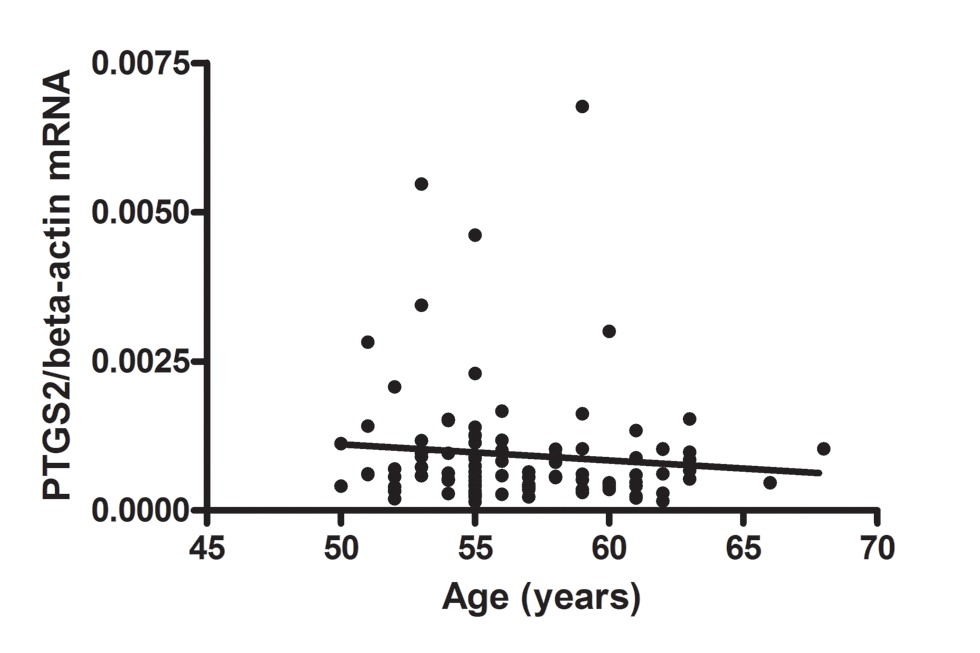

Supplement: Figure S1 — Normalised PTGS2 mRNA levels decreased with age in normal tissue from individuals with dysplasia. (TIF) [file pone.0105254.s001.tif]
